# Geographic Density and Uptake of Pre-exposure Prophylaxis (PrEP) Among Young Gay, Bisexual and Other Sexual Minority Men: A Global Positioning System (GPS) Study

**DOI:** 10.1007/s10461-021-03249-1

**Published:** 2021-04-05

**Authors:** Byoungjun Kim, Basile Chaix, Yen-Tyng Chen, Denton Callander, Seann D. Regan, Dustin T. Duncan

**Affiliations:** 1 Department of Epidemiology, Columbia Spatial Epidemiology Lab, Columbia University Mailman School of Public Health, 722 West 168th Street, New York, NY 10032 USA; 2 Sorbonne Université, INSERM, Institut Pierre Louis d’Epidémiologie et de Santé Publique IPLESP, Nemesis Team, 75012 Paris, France; 3 Chicago Center for HIV Elimination, Chicago, IL 60637 USA; 4 Department of Medicine, University of Chicago, Illinois, 60637 USA

**Keywords:** Pre-exposure prophylaxis, Spatial analysis, Geographic access, Mobility, HIV prevention

## Abstract

The geographic availability of pre-exposure prophylaxis (PrEP) providers is one important factor that significantly affects PrEP uptake. While most previous studies have employed spatial accessibility in static residential neighborhood definitions or self-reported healthcare accessibility, we examined the associations of the objectively measured geographic density of PrEP services with current PrEP use, using global positioning system (GPS) among sexual minority men (SMM) in New York City. 250 HIV-negative SMM participated in a 2-week GPS monitoring (January 2017–January 2018). Geographic PrEP density was measured as total numbers of PrEP providers in (1) individual activity space defined as daily path area of GPS points, (2) residential street network buffers and (3) census tract and ZIP code of residential locations. Geographic PrEP density within GPS-based activity space was positively associated with current PrEP use (prevalence ratio for 50-m activity space = 1.10, 95% confidence interval: [1.02, 1.18]). PrEP provider counts in residential buffer areas and administrative neighborhoods were not associated with PrEP use. Although it is not generalizable beyond New York City, our finding suggests the importance of daily mobility pattern in HIV prevention and PrEP implementation strategies.

## Introduction

Despite a recent decline in human immunodeficiency virus (HIV) infection in the general population in the United States (U.S.), young gay, bisexual and other sexual minority men (SMM) face a disproportionately high burden of HIV. To illustrate, the rate of new HIV diagnoses in 2012 among young SMM aged 13–24 years was more than double the rate in 2002 in the U.S. [1]. In 2016, SMM accounted for 81.0% of newly diagnosed HIV infections among individuals aged 13 to 24 years [2]. Another report estimated that HIV incidence among SMM between age 25 and 34 increased by 5.7% between 2008 and 2015 [3]. In 2012, the U.S. Food and Drug Administration (FDA) approved daily use of tenofovir disoproxil fumarate/emtricitabine (TDF/FTC) for the prevention of sexually-acquired HIV, and it was determined that pre-exposure prophylaxis (PrEP) is an effective biomedical HIV prevention strategy [4–7]. For example, one multi-site randomized trial found that daily PrEP use significantly reduced the risk of HIV infection (Hazard Ratio: 0.56, p-value: 0.005) among SMM and transgender women [4], and the Center for Disease Control and Prevention (CDC) also demonstrated that PrEP reduced the risk of HIV infection by more than 90% among SMM and other vulnerable populations [8].

Since its introduction, PrEP uptake has increased dramatically among all target populations in many parts of the U.S. [9–11], yet, the rate among young SMM between age 18 and 25 remains relatively low, and young SMM tend to have high discontinuation [12–15]. In addition to individual-level factors of PrEP uptake, including concerns about long-term side effects, medical mistrust, or lack of health insurance [16–21], supra-individual structural barriers, such as stigma against PrEP use and limited PrEP availability, have been identified as determinants of PrEP uptake [22–25].

Research has recently started to examine the geography of PrEP providers as another factor of disparities in PrEP acceptability or uptake [26–28]. For example, both state-level and community-level analyses showed positive associations between geographic PrEP availability and awareness/uptake in the U.S. However, most of the studies have been limited by the use of static definitions of administrative residential neighborhood boundaries. A recent review of the role of neighborhood environments on HIV infection among SMM indicated the majority of studies employed residential administrative boundaries, specifically ZIP codes and census tracts [29]. Most of the important contextual data, for instance census data or HIV statistics, are available for the standard administrative definitions, however, such crude residential neighborhood definitions might not fully capture the actual neighborhood-level factors of HIV infection. Such administrative residential neighborhood definitions can only cover a small portion of an individual’s actual activity space which includes different places in daily activities [30, 31]. Activity space, defined as a geographic space where people travel in the course of daily activities, encompasses neighborhoods where individuals visited for various purposes, for example residential, work, and socializing, as well as the travel itineraries of people between these places [32]. In addition, most of the research on geographical analysis of PrEP availability employed ecological study design using aggregated data, which are susceptible to the ecological fallacy.

In the present study, we employed global positioning system (GPS) tracking technology to define the individual-level activity spaces of young SMM in New York City. GPS technology is an objective approach to measure spatial mobility and activity space by allowing investigators to collect data on continuous locations of participants over time. These developments in defining GPS-based activity space have been applied in the field of obesity research and related risk behaviors [33–36], but it has not widely employed in HIV prevention and epidemiology due to the sensitive nature of HIV-related data. In this study, we used this technology to investigate a geospatial association between PrEP providers and PrEP uptake. Although we were aware of the potential issue of selective daily mobility bias [33], we hypothesized that increased number of PrEP providers in individual’s activity space would be associated with a greater likelihood of uptake among our sample, through potential behavioral factors, such as reduced travel time to initiate and continue PrEP use and greater exposures to PrEP advertisements and promoting locations. Further, as we discussed the limitations of previous studies on geography in PrEP and uptake, we sought to compare our study findings based on geospatial techniques with different approaches of defining of residential neighborhoods including conventional census tract and ZIP code.

## Methods

### The P18 Neighborhood Study

This analysis drew upon participants of the *Project 18 Cohort Study*, a prospective cohort study of 665 HIV-negative or unknown status SMM in New York City (NYC), which focused on sexual behavior, substance use, and mental health [37]. To be eligible for this study, participants had to be male, cisgender, aged 18 to 19 years, reside in the NYC metropolitan area, report having had sex with another man in the 6-month period before screening, and be a self-reported negative HIV serostatus or unknown status. The first phase of the study started from June 2009 with 274 participants; between June 2014 and March 2016, an additional 391 participants were recruited if they were 22 to 23 years old and met the same inclusion criteria of the previous phase.

For the more focused subgroup study, known as the *P18 Neighborhood Study* [38], 450 participants were randomly selected and invited to participate in the sub-study via email. In total, 250 participants enrolled in the sub-study, and it was conducted from January 2017 to January 2018. The additional eligibility criteria applied for the subgroup study included being HIV-negative, having no mobility restrictions, being comfortable carrying the GPS device for 2 weeks, and being able to come in for two study visits. At the first visit, participants were consented to the subgroup study and completed the first survey. The first survey included perceptions and experiences in different neighborhoods. Instructions on GPS device and GPS-use diary—daily log of GPS carrying and charging—were also provided at the first visit. At the second visit, they returned the device and GPS-use diary as well as completed a second survey for GPS use acceptability. Of the 250 participants, 3 participants did not complete their study visits, therefore 3 additional participants were enrolled. The University Committee on Activities Involving Human Subjects at New York University approved the research protocol and written informed consent was obtained prior to participation in this study. New York University School of Medicine also approved the research protocol (IRB #: i16-00082).

### GPS Protocol and Activity Space Definition

Participants were instructed to carry a small GPS device (BT-Q1000XT, QStarz International Co., Ltd., Taipei, Taiwan) for 2 weeks in their pocket at all times except sleeping, swimming, and showering. The device was designed to log locations in 10-s intervals, and in order to better understand participants’ recorded GPS data, they were asked to complete a GPS-use diary. A prior pilot study was conducted to examine the feasibility and acceptability of proposed GPS protocols in this population [39], and the similar protocol was used in this study. The GPS data extracted from the devices were processed based on a set of processing scripts to eliminate duplicate time stamps and isolated GPS points (400-m or longer distance between two consecutive points corresponding to a 10 s interval) which were likely data errors.

To define the activity spaces of participants, we employed daily path area (DPA) calculations. DPA is one advanced method in behavioral geography [40–43], which has been shown to accurately capture travel routes and destinations without overgeneralization [44]. The DPA was defined by creating 50-, 100-, 200-, and 400-m dissolved buffering zones around participant GPS points, excluding any records outside of NYC due to limitation of data acquisition for neighborhood-level HIV prevalence. In addition, the current PrEP use measure of the P18 study specifically asked whether the participants took PrEP from providers in NYC. All GPS data processing and cleaning were conducted using ESRI ArcGIS 10.4, and Quantum QGIS 2.6.

**Fig. 1:**
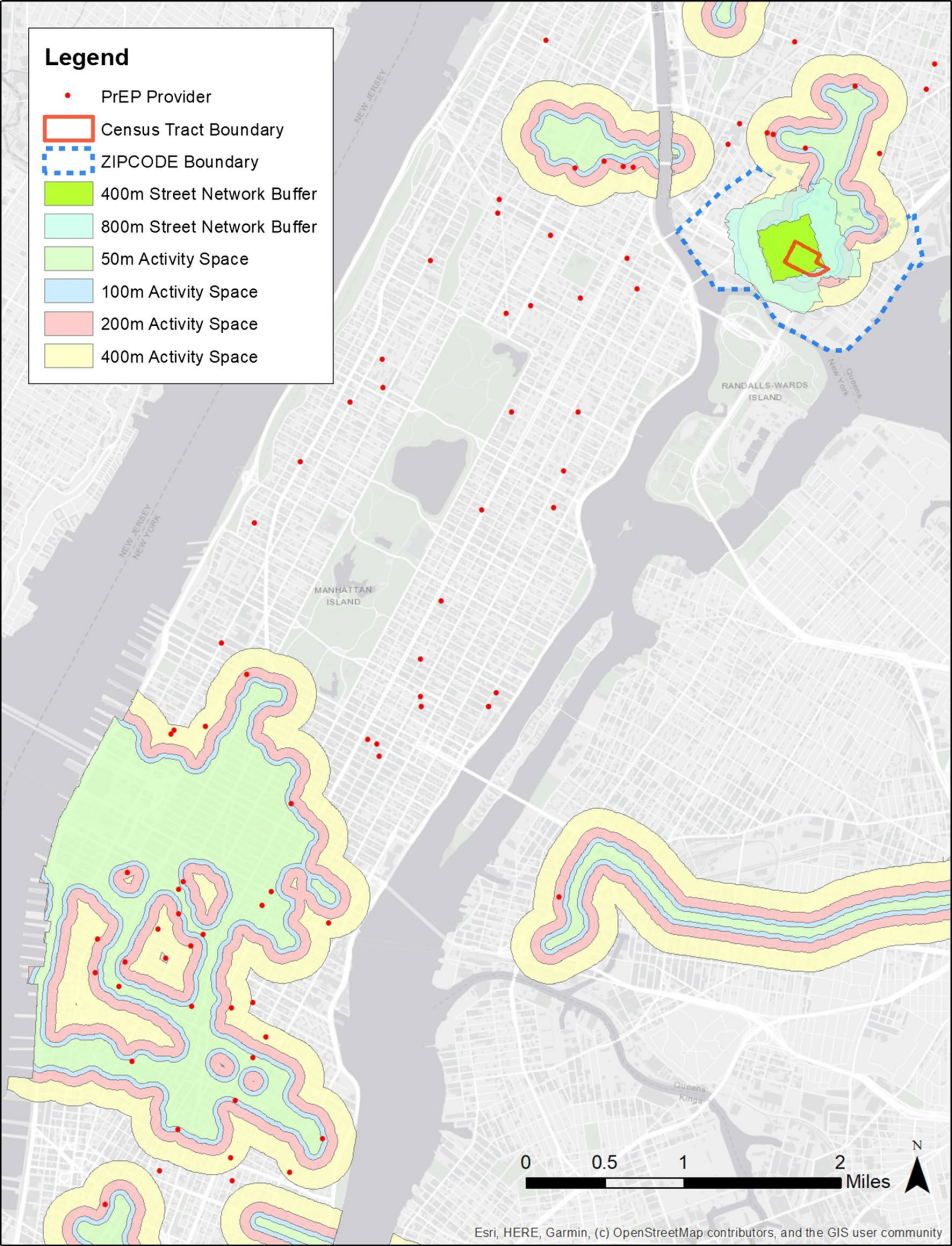
Example of GPS-based activity space and residential administrative boundaries

Of 250 total participants with GPS data, 39 participants were excluded because they lived outside of NYC (n = 26), had invalid addresses (n = 9) and/or had less than 1 h of GPS data for each day (n = 4), and a total of 211 participants were used for the analysis. An example map of one participant’s activity space, residential area, and surrounding PrEP provider locations is provided in Fig. 1.

### Geographic PrEP Density

Data on PrEP prescribing clinics and healthcare professionals were obtained from the Centers for Disease Control and Prevention National Prevention Information Network (CDC NPIN) [45]. The PrEP Locator Database is a national database of PrEP-prescribing clinics, hospitals, and organizations, providing a unified and vetted source of PrEP providers across the U.S. The data collection process included web searches, referrals, and outreach processes to state health departments, and the dataset was consistently reviewed by an advisory board and staff of CDC NPIN [46]. By October 2018, there were 154 registered PrEP providers in NYC, and we geocoded the addresses using the ArcGIS Online Geocoding Service.

Geographic PrEP density was calculated based on the number of geocoded PrEP provider locations in GPS-based activity space definitions. Total numbers of PrEP services located in 50, 100, 200, and 400 m buffered activity spaces were calculated. For comparison, the number of PrEP locations were also calculated using conventional neighborhood definitions, including street network buffers (400 m and 800 m radius), ZIP code and census tract of residential locations. All geographic calculations were performed on ESRI ArcGIS 10.4.

### PrEP Use

One dichotomously coded question was used to determine current PrEP use: “Are you currently using daily oral PrEP available in NYC?” When answering this question, participants were provided with the following definition of PrEP: “an HIV-negative person taking a daily pill to prevent HIV”. Additionally, we also examined lifetime PrEP use from a question: “Have you ever taken PrEP?”.

### Covariates

Participants reported their socio-demographic characteristics. Age, ethnicity, race, level of education, current school enrollment status, annual income and foreign-born status were included in the analyses. The survey did not include employment status or occupation type which may be important factors of mobility, but given the relatively young ages of participants, the school enrollment status and annual income may adequately capture the association between employment/occupation and mobility [47]. Participants were also asked about their residence/housing type (family apartment/house, own apartment/house, living with friends/roommates, dorm/residence hall/school housing, single room occupancy, hostel, temporary housing/shelter or other), as housing type is a salient risk factor of HIV infection [48]. Self-reported sexual identity, number of sexual partners in the past 6 months, and current relationship status (currently had a main romantic partner) were included in the analysis, as they are important predictors of PrEP use [19]. Lastly, residential self-selection of individuals, referring individuals’ propensity to choose where to live based on their life needs and preferences, may influence the mobility patterns and health behaviors [49, 50]. In this case, an ordinal variable that measured importance of living in “gay” neighborhood in Likert scale (e.g. not at all important, not too important, somewhat important, mostly important, and very important) was included, as “gay” neighborhood is associated with availability of PrEP prescribing services [51], and social group membership and increased social capital may positively affect PrEP uptake [52].

Census-tract level sociodemographic characteristics were retrieved from the 2017 U.S. Census American Community Survey [53]. The neighborhood sociodemographic characteristics included the percentage of Hispanic and non-Hispanic black residents, the percentage of people who lived below federal poverty level, and the percentage of same-sex couple households as a proxy of gay population. To calculate the sociodemographic characteristics in individual’s activity space, we employed an area-weighted average which allocates proportions of each census-tract dependent on the overlapped area with the individual’s activity space and calculates weighted average based on the proportions. For example, assuming that each census tract A, B, C, and D respectively corresponded to 20%, 30%, 15%, and 35% of the daily path area, we calculated the value of the sociodemographic variable for the daily path area as a sum of products: 0.2*V_a_ + 0.3*V_b_ + 0.15*V_c_ + 0.35*V_d_ where V_a_ ~ V_d_ are the values of the variable (e.g. percentage of Black) for each census tract.

Lastly, area-weighted average of HIV prevalence was constructed in the same way as neighborhood socio-demographics, using ZIP code tabulated area (ZCTA)-level HIV prevalence data which was the smallest geographic unit of HIV prevalence data. The data were obtained from the NYC Department of Health and Mental Hygiene (NYC DOHMH) via AIDSVu [54, 55]. The number of people living with HIV/AIDS (PLWHA) in NYC represents people diagnosed with HIV/AIDS, reported to the NYC DOHMH as of September 30, 2017 and presumed to be living as of December 31, 2016.

### Statistical Analyses

Descriptive statistics were calculated and are presented in Table 1. To test associations between geographic PrEP density and PrEP use, we employed log-binomial regression using an adaptive barrier algorithm to estimate prevalence ratios [56–59]. We examined bivariate models as well as fully adjusted multivariate models with PrEP provider counts in individual’s activity space as the main outcome variable. In addition, the results from activity space measures were compared with additional models in which conventional neighborhood definitions were used, and the same individual and neighborhood-level covariates were used as in the activity space models. We also tested multilevel models with random effects for the 5 boroughs as well as the 211 ZIP codes in New York City, but the results indicated that there were no between-cluster variances (i.e. intraclass correlation coefficient), thus the standard multivariable model results were reported. Lastly, we tested a set of sensitivity analyses using the sizes of activity spaces (in km^2^) and distance travelled as exposure variables with the same covariates to examine whether the associations were potentially due to the greater mobility of certain participants. All statistical analyses were conducted using R.3.3.2.

**Table 1:** Descriptive statistics of individual variables, the P18 Neighborhood Study (n = 211)

Variables	Mean (SD) or N (%)
Age (years, min = 23, max = 26)	24.9 (0.9)
Sexual identity	
Gay	177 (84)
Bisexual	30 (14)
Others	4 (2)
Race	
White	67 (32)
Black/African American	64 (30)
Asian	21 (10)
Others	35 (17)
Two or more	21 (10)
Ethnicity	
Non-Hispanic/Latino	148 (70)
Hispanic/Latino	63 (30)
Annual income (missing = 14)	
< $15,000	51 (24)
$15,000–$35,000	70 (33)
> $35,000	76 (36)
Current student (yes)	52 (25)
Education (missing = 1)	
≤ High School	71 (34)
Associate	23 (11)
College/Graduate	116 (55)
Current housing	
Family housing	68 (32)
Own housing	27 (27)
Friends/Roommates	71 (34)
Others	13 (6)
Foreign-born (yes)	30 (14)
Importance of “gay” neighborhood in current housing choice (missing = 12)	
Not at all important	65 (31)
Not too important	74 (35)
Somewhat important	40 (19)
Mostly important	7 (3)
Very important	13 (6)
Currently has a romantic partner (yes, missing = 34)	77 (36)
Number of sexual partners (past 6 months)	5.8 (7.4)
Current PrEP use (yes, missing = 6)	33 (16)
Lifetime PrEP use (yes, missing = 6)	58 (27)

*SD* Standard deviation

## Results

Table 1 shows individual characteristics of the P18 Neighborhood Study participants. Briefly, the participants were relatively diverse in terms of race and ethnicity, and 25% were current students. Thirty-three out of 211 participants were currently taking daily oral PrEP from providers in NYC (16%). Descriptive statistics of neighborhood variables are provided in Table 2. The average number of PrEP providers in activity spaces, as expected, increased with larger buffer sizes of DPA (see Fig. 1). Further, the underlying sociodemographic characteristics of the population in the areas changed significantly depending on the neighborhood definitions we employed. For example, the percentages of black in participants’ residential census tract and ZIP code had mean values of 31% and 29%, respectively, whereas it was around 18% in area weighted GPS-based activity space definitions (Table 2). Also, the percentage of people who lived below poverty level was higher when using residential census tract or ZIP code, and the percentage of same-sex couple households was higher in activity space definitions than residential definitions (Table 2). These differences between the GPS-based activity space definitions and residential areas suggest that the participants traveled to neighborhoods with lower percentages of Black and people living in poverty, as well as visited neighborhoods with comparatively higher proportions of same-sex couples living there.

**Table 2:** Descriptive statistics of neighborhood variables, the P18 Neighborhood Study (n = 211)

	GPS-based Activity Space [mean, (SD)]				Residential Area [mean, (SD)]			
	50 m Buffer	100 m Buffer	200 m Buffer	400 m Buffer	400 m Network	800 m Network	Census Tract	Zip Code
PrEP Provider Count	7.0 (5.1)	9.4 (6.4)	14.8 (9.5)	24.9 (14.5)	0.2 (0.5)	1.0 (1.4)	0.1 (0.4)	1.4 (1.4)
Mean size (km^2^)	6.0 (5.1)	9.4 (8.2)	16.3 (14.1)	29.2 (24.3)	0.29 (0.03)	1.16 (0.13)	0.36 (0.74)	4.25 (4.06)
% Black	18.2 (12.2)	18.1 (11.7)	17.8 (11.0)	17.6 (10.1)	23.9 (20.6)	27.1 (18.8)	31.4 (27.1)	29.2 (24.1)
% Hispanic	23.9 (11.0)	24.1 (10.9)	24.2 (10.7)	24.1 (10.2)	40.9 (19.5)	37.1 (16.3)	32.2 (22.6)	33.2 (22.6)
% Poverty	18.4 (5.3)	18.4 (5.2)	18.4 (5.0)	18.4 (4.8)	25.6 (9.5)	25.1 (8.0)	23.6 (12.2)	23.3 (12.2)
% Same-sex households	0.93 (0.49)	0.90 (0.47)	0.88 (0.45)	0.87 (0.42)	0.44 (0.52)	0.42 (0.39)	0.79 (1.00)	0.73 (1.01)
HIV Prevalence (/100,000)	2418 (819)	2375 (793)	2337 (760)	2291 (701)	2236 (1105)	2203 (1032)	2285 (1171)	2285 (1171)

The crude and adjusted associations between geographic PrEP density and PrEP use are shown in Table 3. The adjusted prevalence ratios (PRs) of current PrEP use for all activity space definitions showed positive associations (50 m-buffer activity space: PR 1.10, CI [1.02–1.18]; 100 m: PR 1.07, CI [1.01–1.14]; 200 m: PR 1.06, CI [1.00–1.11]; 400 m: PR 1.04, CI [1.01–1.08]). To illustrate, the prevalence of current PrEP use was 10% higher with each additional PrEP provider in the 50 m radius activity space among young SMM participants. When using residential definitions of geographical PrEP density, there were no associations (400 m street network buffer: PR 0.51, CI [0.13–2.01]; 800 m street network buffer: PR 0.80, CI [0.45–1.42]; census tract boundary: PR 0.39, CI [0.07–2.12]; ZIP code: PR 1.72, CI [0.74–1.85]). The PRs of lifetime PrEP use for activity space definitions showed no or weak associations, and again, the residential definitions had no associations with lifetime PrEP use (Table 3). Lastly, the sensitivity analyses with sizes (i.e. areas) of activity spaces (in km^2^ for 50-, 100-, 200-, and 400-m buffers) and distance travelled (in km) as additional exposure variables showed no associations between those mobility measures and PrEP use (data now shown).

**Table 3:** Associations between geographic PrEP access and PrEP use (N = 211)

PR (CI)	PrEP Provider Count in Activity Space				PrEP Provider Count in Residential Area			
	50 m Buffer	100 m Buffer	200 m Buffer	400 m Buffer	400 m Buffer	800 m Buffer	Census Tract	ZIP Code
Lifetime PrEP Use								
Crude PR	1.03 (1.00, 1.06)	1.03 (1.00, 1.06)	1.01 (1.00, 1.03)	1.01 (1.00, 1.02)	1.40 (0.94, 2.10)	1.05 (0.86, 1.27)	1.53 (0.79, 2.95)	0.93 (0.76, 1.14)
Adjusted PR	1.04 (0.98, 1.11)	1.05 (1.00, 1.10)	1.03 (1.00, 1.06)*	1.02 (1.00, 1.04)	1.79 (0.99, 3.24)	1.02 (0.80, 1.31)	1.72 (0.99, 2.98)	0.93 (0.74, 1.17)
Current PrEP Use								
Crude PR	1.05 (1.01, 1.10)*	1.05 (1.01, 1.08)*	1.02 (1.00, 1.05)	1.01 (1.00, 1.03)	0.49 (0.15, 1.65)	0.71 (0.45, 1.11)	0.37 (0.06, 2.51)	0.88 (0.65, 1.20)
Adjusted PR	1.10 (1.02, 1.18)*	1.07 (1.01, 1.14)*	1.06 (1.00, 1.11)*	1.04 (1.01, 1.08)*	0.51 (0.13, 2.01)	0.80 (0.45, 1.42)	0.39 (0.07, 2.12)	1.72 (0.74, 1.85)

Adjusted for individual-level age, sexual identification, race, ethnicity, income, education, student status, housing type, “gay” neighborhood as residential self-selection, foreign born status, relationship type, and number of sexual partners as well as neighborhood-level percentage black, Hispanic, poverty, same-sex couple households, HIV prevalence

*PR* Prevalence Ratio, *CI* Confidence interval

*P-value < 0.05

## Discussion

This study examines geographic PrEP access (i.e. PrEP density) using different neighborhood definitions, and we demonstrate that current PrEP use was positively associated with more PrEP providers in the vicinity, only when the geographic density was measured with GPS-based activity space definitions (PR for one provided increase 50-m activity space = 1.10, 95% confidence interval: [1.02, 1.18]). The associations are consistent across different buffer sizes of DPA (50, 100, 200 and 400 m), and interestingly, the association is not detected from models considering access in conventional residential areas. Consistent with previous individual-level studies that have identified the ease of physical access to PrEP providers as a facilitator of PrEP uptake [23, 60], our findings support the notion that activity spaces can contribute to the measurement of geographic proximity to PrEP providers. As a few studies has identified that geographic accessibility is an important factor influencing PrEP uptake [61, 62], we demonstrated that current PrEP use is associated with PrEP provider density within activity space. The probability of current PrEP use was 10% higher for each additional provider in the participants’ GPS track buffer (50 m radius) and this association should be interpreted in light of the distribution of the number of PrEP providers in the 50 m DPA (median: 4.68, 10^th^ and 90^th^ percentiles: 1.92, 11.09). It may indicate that initiating or continuing PrEP prescription may be a less burden when there are more providers in the vicinity, and having more PrEP providers in individual’s activity space may reduce the burden of travels for office visits as well as increase exposures to PrEP promoting services. Additionally, the differences in results between activity space definitions and residential areas are consistent with other GPS-based findings on food environments and diet. For example, Zenk et al. found no associations between neighborhood features in residential neighborhoods and obesity-related behaviors, but some measures based on GPS activity space definitions (e.g. fast food restaurant or supermarket density in activity space) were associated with dietary behaviors [63]. This may imply that the characteristics of the residential areas itself may not be an adequate measure of social and environmental exposures in behavioral geography and public health research.

This study is not without limitations. First, the study was conducted in New York City, and the participants were sampled from HIV-negative young SMM. Thus, our findings may not be generalizable to other environments, such as small cities and rural areas, and other SMM subpopulations, such as HIV-positive SMM or older SMM. Second, although the 2-week GPS monitoring period is relatively long compared to most health studies using GPS [40], the activity space assessed over the period may not represent participants’ typical travel behaviors. However, a recent study by Zenk et al. reported that a 2-week period is adequate to measure representative activity space [64]. Third, the GPS protocol was designed for 2-week data collection, but not all of participants allowed the full-period tracking. We did not standardize the activity space size by number of days of tracking, however such standardization is not methodologically straightforward. Fourth, in addition, the GPS device utilizes GPS satellites by continuously receiving geolocation and time information, however, GPS signal errors and data losses may be introduced due to special settings in large metropolitan locations. Subway, underground environment, and large buildings could create so-called “street canyons” which block the communications with satellites [65]. To address the GPS error, we processed the data to maximize reliability by eliminating isolated points and duplicated timestamps. Moreover, the GPS signal loss in the subway transit system is not likely to bias the assessment of actual proximities to PrEP providers. Fifth, there was a temporal mismatch between the datasets used. The PrEP use questionnaire was collected in 2017, while the PrEP Locator data were retrieved as of October 2018. Also, the HIV prevalence data was from 2016 surveillance, and the socio-demographics were 5-year estimates between 2012 and 2016. Lastly, the potential selective daily mobility bias, referring potential bias due to the fact that participants’ travel behavior and route were selected based on preferences related to PrEP use, might have contributed to the associations reported in the present study [33, 66]. The GPS-based measure of density of PrEP providers may reflect personal behavior of visiting PrEP providers, if participants intentionally visited a provider during the GPS monitoring period. In this case, the measure of spatial access is flawed, and the association of interest is driven by a priori willingness to use PrEP. However, considering the fact that the regular office visit for PrEP prescription is usually every 3 months, the 2-week GPS-measured spatial access may not be significantly affected by the selective daily mobility bias. Additionally, it is possible that participants who had more travels shared similar cultural norms and personalities, such as impulsivity or sensation-seeking, that promote/barrier PrEP uptake [67]. We tested sensitivity analysis using participants’ activity space sizes and distance travelled during the GPS monitoring period and found no associations with PrEP use, which also suggests that the association was not identified to certain participants being more mobile but indeed to the differential number of providers in the vicinity of the GPS tracks. Also, such potential biases may be ruled out by adjusting the presence of romantic partner in the analysis.

Our study has numerous strengths, including assessing the actual extent of individual’s activity space as an objective measure, a large sample size for a sensor-based study, and a relatively long period of GPS tracking [40, 64]. To our knowledge, this study is the largest GPS study to examine the association of geographic PrEP provider density with PrEP use. The GPS protocol allowed 10-s epoch, which is a high monitoring frequency that enhanced the overall quality of the GPS data.

These findings motivate additional research to better understand individual activity space and local mobility for effective HIV prevention. Our findings suggest that locations frequently visited by target populations, other than their residential areas, should be considered as potential intervention locations when planning additional PrEP providers in NYC. The associations of geographic PrEP provider density in activity space with PrEP uptake suggest that future analyses on identifying clusters of overlapping activity spaces of young SMM may help identifying potential target areas for PrEP implementation. These analyses may raise additional complexity in handling temporal components of the activity space, but coupled with the findings, such sophisticated clustering analyses may enable to optimize the targeting of intervention strategies by time, day, and space. The future research may employ a novel method of GPS tracking combined with ecological momentary assessment (EMA) to better understand the actual exposures to HIV-related environments in conjunction with real-time measures of participants’ feelings, perceptions and specific behavior (e.g., sexual behavior) [68].

Our findings can guide future PrEP implementation strategies. A previous study investigated HIV diagnoses in residential neighborhoods in relation to PrEP provider density in NYC and noted that ZIP codes with high rates of HIV infections had larger numbers of PrEP clinics [51]. Beyond the at-risk areas based on residence, which can be considered as an indicator of places needing intervention, it would further enhance the effectiveness of HIV prevention programs to consider activity spaces and travel patterns of populations at risk for HIV when defining where to intervene. In addition, young SMM who experience difficulties in PrEP adherence and have higher PrEP discontinuity may benefit from additional PrEP access points in places where they tend to cluster during their daily activities.
